# The Role of Gut Microbiota and Environmental Factors in Type 1 Diabetes Pathogenesis

**DOI:** 10.3389/fendo.2020.00078

**Published:** 2020-02-26

**Authors:** Sandra Dedrick, Bharathi Sundaresh, Qian Huang, Claudia Brady, Tessa Yoo, Catherine Cronin, Caitlin Rudnicki, Michael Flood, Babak Momeni, Johnny Ludvigsson, Emrah Altindis

**Affiliations:** ^1^Biology Department, Boston College, Chestnut Hill, MA, United States; ^2^Department of Clinical and Experimental Medicine, Linköping University, Linköping, Sweden

**Keywords:** microbiome, longitudinal studies children, type 1 diabetes, environmental factors, autoimmunity

## Abstract

Type 1 Diabetes (T1D) is regarded as an autoimmune disease characterized by insulin deficiency resulting from destruction of pancreatic β-cells. The incidence rates of T1D have increased worldwide. Over the past decades, progress has been made in understanding the complexity of the immune response and its role in T1D pathogenesis, however, the trigger of T1D autoimmunity remains unclear. The increasing incidence rates, immigrant studies, and twin studies suggest that environmental factors play an important role and the trigger cannot simply be explained by genetic predisposition. Several research initiatives have identified environmental factors that potentially contribute to the onset of T1D autoimmunity and the progression of disease in children/young adults. More recently, the interplay between gut microbiota and the immune system has been implicated as an important factor in T1D pathogenesis. Although results often vary between studies, broad compositional and diversity patterns have emerged from both longitudinal and cross-sectional human studies. T1D patients have a less diverse gut microbiota, an increased prevalence of Bacteriodetes taxa and an aberrant metabolomic profile compared to healthy controls. In this comprehensive review, we present the data obtained from both animal and human studies focusing on the large longitudinal human studies. These studies are particularly valuable in elucidating the environmental factors that lead to aberrant gut microbiota composition and potentially contribute to T1D. We also discuss how environmental factors, such as birth mode, diet, and antibiotic use modulate gut microbiota and how this potentially contributes to T1D. In the final section, we focus on existing recent literature on microbiota-produced metabolites, proteins, and gut virome function as potential protectants or triggers of T1D onset. Overall, current results indicate that higher levels of diversity along with the presence of beneficial microbes and the resulting microbial-produced metabolites can act as protectors against T1D onset. However, the specifics of the interplay between host and microbes are yet to be discovered.

## Introduction

The human microbiome consists of trillions of bacterial, viral, and fungal microorganisms (1). They have symbiotically co-evolved with humans for hundred thousands of years (2). The extensive diversity of these microorganisms results from host lifestyle, geographical location, infections, diet, sex, age, and genetic background (3–6). It is shown that the human microbiome can affect many aspects of host physiology, such as metabolism, immunity, and behavior (7–9). Therefore, the disruption of human microbiota could result in disease. A plethora of studies have demonstrated that the human microbiome has the potential to affect the pathogenesis of immune diseases, particularly autoimmune diseases in which the immune system fails to distinguish self from non-self proteins and attacks self-tissues. Examples of such diseases include multiple sclerosis (8), rheumatoid arthritis, systemic lupus erythematosus, anti-phospholipid syndrome (10), Crohn's disease (11–13), ulcerative colitis (14), inflammatory bowel diseases (15, 16), coeliac disease (17), and Type 1 Diabetes (T1D) (18, 19).

The presence of specific gut microbiota, along with overall diversity, are critical in the development of the nascent immune system. Studies conducted in germ-free (GF) and gnotobiotic mouse models reveal their important role in the modulation and differentiation of innate immune cell-types, most notably IL-17 producing CD4+ T cells (Th17) and Foxp3+ T regulatory cells (Tregs) (20, 21). Specifically, segmented filamentous bacteria (SFB) are known to induce the expression of pro-inflammatory Th17 cells, important for maintaining the mucosal barrier, and protect NOD mice from developing type 1 diabetes (20, 22, 23). However, in other mouse models of autoimmune disease (e.g., K/BxN mouse model of autoimmune arthritis), SFB are shown to promote disease progression via increased Th17 accumulation, suggesting their role in autoimmunity is etiologically-specific (24). Other protective bacteria, such as Lactobacilli, Bifidobacteria and *Clostridium* species, are implicated in the induction of anti-inflammatory Treg cells while *Bacteroides fragilis* polysaccharide A (PSA) induces IL-10 production and is known to suppress Th17 cell responses (25, 26).

While the pathogenesis of autoimmune disease remains largely unknown, genetic predisposition (27, 28) and environmental factors (29, 30) have been suspected as major causes. Among environmental factors, the microbiome is correlated with autoimmune diseases via direct and indirect interactions with innate and adaptive immune cells. This results in loss of immune tolerance, chronic inflammation, and immune response against host tissues (31–33). The proposed mechanisms of the microbiome's role in autoimmunity include molecular mimicry, epitope spreading, bystander activation, and prolonged infection (32, 34, 35). Therefore, the loss of immune tolerance might be induced by alteration of microbiome composition. Several studies have, indeed, shown that the gut microbiome composition of individuals with autoimmune diseases is significantly different from those of healthy controls (36–41).

With a few exceptions (e.g., Sardinia, Italy), autoimmune disease incidence follows a north-south trajectory, with higher incidences found in Nordic countries, such as Finland, Sweden and Norway (42, 43). Gut microbiota studies on the effect of extreme climate conditions (i.e., polar expeditions) (44) and birth month/place (45, 46) illustrate how climate, particularly sunlight exposure, affects microbiota composition and immune dysregulation. Factors prominent in northern populations—circadian rhythm disruption and vitamin D deficiencies—have also been shown to result in immune dysregulation via shifts in gut microbiota, leading to autoimmune diseases like T1D (47–49).

Type 1 diabetes (T1D) is regarded as a disease characterized by insulin deficiency resulting from autoimmune destruction or loss of function of pancreatic β-cells. It is important to note that although autoimmunity is seen as the main factor causing T1D by a majority of scientists, some scientists think that autoimmunity is secondary to other factors including endoplasmic reticulum stress leading to beta cell loss (50). Historically, the diagnosis of T1D has considered elevated blood glucose levels (hyperglycemia), and the presence of one or more autoantibodies, all of which occur/are present prior to β-cell ablation (51). Autoimmunity can be mounted against insulin (IAA), glutamic acid decarboxylase (GADA), insulinoma-associated autoantigen 2 (IA2A) and/or zinc transporter 8 (ZnT8A) and may occur many years prior to symptom onset (51). In addition to these main autoantibodies, a newly discovered family of neoepitopes formed with post-translational modification were identified (52, 53). Hybrid insulin peptides (HIPs) are one of the most interesting neopeptides that are formed with the fusion of an insulin peptide with a secretory granule peptide in the granules (54). The most common autoantibody observed prior to disease onset is against IAA, with IAA concentrations correlating strongly with the rate of progression to overt T1D in children (51). This process is commonly referred to as seroconversion, an important term found throughout this review.

While T1D is one of the most commonly represented chronic diseases in childhood, around 25% of people diagnosed with the disease are adults (55, 56). Globally, the incidence of T1D has been increasing for several decades; specifically, after World War II in the western world (57–59). Despite the significant genetic influence, the rise of T1D prevalence and variable incidence rates across different countries, and even between some neighboring areas in Europe (e.g., Russian Karelia and Finnish Karelia), implies an interplay between predisposition genes and environmental influences (60, 61). Discordant results for T1D in identical twin studies (30–60% concordant rate), along with migrant studies demonstrating that second generation immigrants to Sweden have an increased risk of developing T1D, all suggest that environmental affects likely play a significant role in disease pathogenesis (62–64).

The non-obese diabetic (NOD) mouse model is the most widely used animal model for the study of T1D due to significant similarities with human T1D in terms of autoantigen recognition, immunopathology, and gene susceptibility (65). Similar to humans, the NOD mouse model displays T1D-associated variance in MHC class II which effects the presentation of islet-derived antigen to T cells. Additionally, multiple non-MHC susceptible genes common to both NOD mice and humans are associated with T1D risk including PTPN22, CTLA-4, IL2RA (66). It was initially observed that colony hygiene status impacts T1D incidence in NOD mice (67). Other NOD mice studies have also demonstrated that the gut microbiome can interact with the immune system to regulate diabetes pathogenesis in mice (68). However, unlike humans, female NOD mice have significantly higher T1D incidence than male NOD mice. A study by Markle et al. (69) found that male NOD mice under specific-pathogen-free (SPF) conditions were more protected against T1D than female SPF mice. However, in germ-free conditions, both male and female mice had equal incidence rates. Certain commensal bacteria known to elevate testosterone levels are also found to be capable of protecting male NOD mice against T1D onset (69). Interestingly, transferring of the gut microbiota from male NOD mice to female mice altered the composition of gut microbiome in recipient mice and, consequently, protected them from T1D (69). This implies that alterations of the gut microbiome can influence immune system and disease pathogenesis.

Despite the apparent differences in NOD mouse model T1D etiology, *in vivo* studies offer a mechanistic approach not possible in human studies and have proven vital in understanding disease onset and progression. In this review, we will discuss the findings from both human longitudinal studies and NOD mice studies and the role of gut microbiota as it relates to environmental factors in type 1 diabetes pathogenesis.

## Human Longitudinal T1D Studies

Genome-wide association studies have identified ~50 genetic regions that affect the risk of developing T1D (70). One's risk for developing T1D is initially determined by identifying genetic factors, such as T1D-associated single nucleotide polymorphisms (SNPs) in the human leukocyte antigen (HLA) gene; more specifically, the HLA-DQ and HLA-DR protein-coding genes DQA1 and DQB1 (71, 72). However, as previously described, genetic predisposition alone is not sufficient to explain the onset of T1D. Cross-sectional studies have successfully identified several environmental factors that might be potential triggers for disease onset (73). Thus far, results from such studies have been broad and primarily correlative, alluding to the multi-faceted nature of the disease.

To identify the causative environmental triggers of disease onset, longitudinal studies of large at-risk cohorts are required across a wide geographical range. Such efforts have been initiated and include: TEDDY, DIABIMMUNE, BABYDIET (a substudy of the larger BABYDIAB), ABIS, TRIGR, and FINDIA (a sub-study with in the Type 1 Diabetes Prediction and Prevention-DIPP Study). In the following section, we will discuss the main findings of TEDDY, DIABIMMUNE, and ABIS, focusing on the role of gut microbiome in the development of autoimmunity. Due to the diet intervention used in BABYDIET, TRIGR, and FINDIA, these longitudinal studies will be discussed in the Diet section of this review.

### Teddy Study

The Environmental Determinants of Diabetes in the Young (TEDDY) study was designed to monitor children identified as at-risk for the development of T1D. The study was conducted at six different clinical centers in Colorado, Washington State, Georgie/Florida in the United States and Finland, Germany and Sweden. Between 2004 and 2010, general population (GP) and first-degree relative (FDR) newborns were screened for human leukocyte antigen (HLA) types (72). For all six geographical regions, the same high-risk haplogenotypes were identified; four of these haplogenotypes were considered for the GP inclusion criteria (DR3/4, DR4/4, DR4/8, DR3/3) while nine haplogenotypes were considered for FDR (DR3/4, DR4/4, DR4/8, DR3/3, DR4/4b, DR4/1, DR4/13, DR4/9, and DR3/9) (https://teddy.epi.usf.edu/research).

Two additional studies were recently conducted using TEDDY samples. In Vatanen et al. (19), stool sample metagenomes were collected and sequenced from 783 children monthly from age 3 months until the clinical end-point (seroconversion, detection of autoantibodies). In islet autoimmunity (IA) case-control cohorts, IA had a higher prevalence of *Streptococcus* group *mitis/oralis/pneumoniae* while controls had higher abundances of *Lactobacillus rhamnosus* and *Bifidobacterium dentium*; two commonly used species in probiotic cocktails (Supplementary Table 1). In T1D case-control cohorts, T1D cases had a higher abundance of *Bifidobacterium pseudocatenulatum, Roseburia hominis*, and *Alistepes shahii* while healthy controls had a higher prevalence of *Streptococcus thermophilus* and *Lactococcus lactis* (Table 1, Supplementary Table 1c). In another study, Stewart et al. (74) analyzed stool samples from 903 children between 3 months and 46 months of age using 16s rRNA (V4) and metagenomic sequencing data. A nested case-control analysis showed that alpha diversity, microbiota maturation and microbiota-by-age Z-scores (MAZ) were similar between cases and controls for both IA and T1D cohorts. The relative abundance of the most abundant genera showed subtle compositional alterations including a higher prevalence of an unclassified *Erysipelotrichaceae* in IA cases (Supplementary Table 1a). Compared to healthy controls, T1D cases had a higher abundance of *Parabacteroides* (*P* < 0.001) and a decreased prevalence of 11 genera including four unclassified *Ruminococcaceae, Lactococcus, Streptococcus, and Akkermansia* (Table 1). Overall, TEDDY identified several bacteria weakly correlate with T1D onset, however, further studies are needed in order to dissect the mechanisms at play.

**Table 1:** Taxa with increased/decreased abundance in T1D subjects identified in longitudinal human studies. Summary of findings from all longitudinal human studies discussed in this review.

**Phylum**	**Class**	**Family**	**Genus**	**Species**	**Study cohort**	**References**
**A.TAXA IDENTIFIED IN LONGITUDINAL STUDIES AS INCREASED IN T1D PATIENTS**						
Actinobacteria	Actinobacteria	Bifidobacteriaceae	*Bifidobacterium*	*pseudocatenulatum*	TEDDY	(19)
Firmicutes	Clostridia	Lachnospiraceae	*Roseburia*	*hominis*	TEDDY	(19)
Bacteroidetes	Bacteroidia	Rikenellaceae	*Alistepes*	*shahii*	TEDDY	(19)
Bacteroidetes	Bacteroidales	Porphyromonadaceae	*Parabacteroides*		TEDDY	(74)
Firmicutes	Bacilli	Streptococcaceae	*Streptococcus*		DIABIMMUNE	(75)
Firmicutes	Clostridia	Lachnospiraceae	*Blautia*		DIABIMMUNE	(75)
Firmicutes	Clostridia	Ruminococcaceae	*Ruminococcus*		DIABIMMUNE	(75)
Bacteroidetes	Bacteroidia	Bacteroidaceae	*Bacteroides*		DIABIMMUNE, FINDIA	(76, 77)
**B.TAXA IDENTIFIED IN LONGITUDINAL STUDIES AS DECREASED IN T1D PATIENTS**						
Firmicutes	Clostridia	Ruminococcaceae			TEDDY	(74)
Firmicutes	Bacilli	Streptococcaceae	*Lactococcus*		TEDDY	(74)
Firmicutes	Bacilli	Streptococcaceae	*Streptococcus*		TEDDY	(74)
Verrucomicrobia	Verrucomicrobiae	Verrucomicrobiaceae	*Akkermansia*		DIABIMMUNE	(75)
Firmicutes	Negativicutes	Veillonellaceae			DIABIMMUNE	(75)

### Diabimmune Study

In northern Europe, highly disparate incidence rates for autoimmune disease have been documented between neighboring countries. For example, although the frequency of HLA genotypes are very similar between Finland and Russian Karelia, the incidence rates of T1D are 6-fold higher in Finland (78). These observed trends are thought to be dependent upon national public health standards and personal hygiene practices; with “developed” nations carrying a higher incidence rate for autoimmune diseases compared to their “less developed” counterparts. Hygiene and autoimmunity (i.e., hygiene hypothesis) are purportedly linked by the observed decrease in gut microbiota diversity in more hygienic conditions (79–81). The loss of alpha diversity is thought to increase the risk of pathogenic invasion (e.g., antibiotic-induced *Clostridium difficile* infections) (82) and has been shown to exacerbate autoimmunity in predisposed individuals. The DIABIMMUNE longitudinal study sought to identify environmental factors that could be attributed to the higher incidence of autoimmune and allergenic diseases.

**Figure 1:**
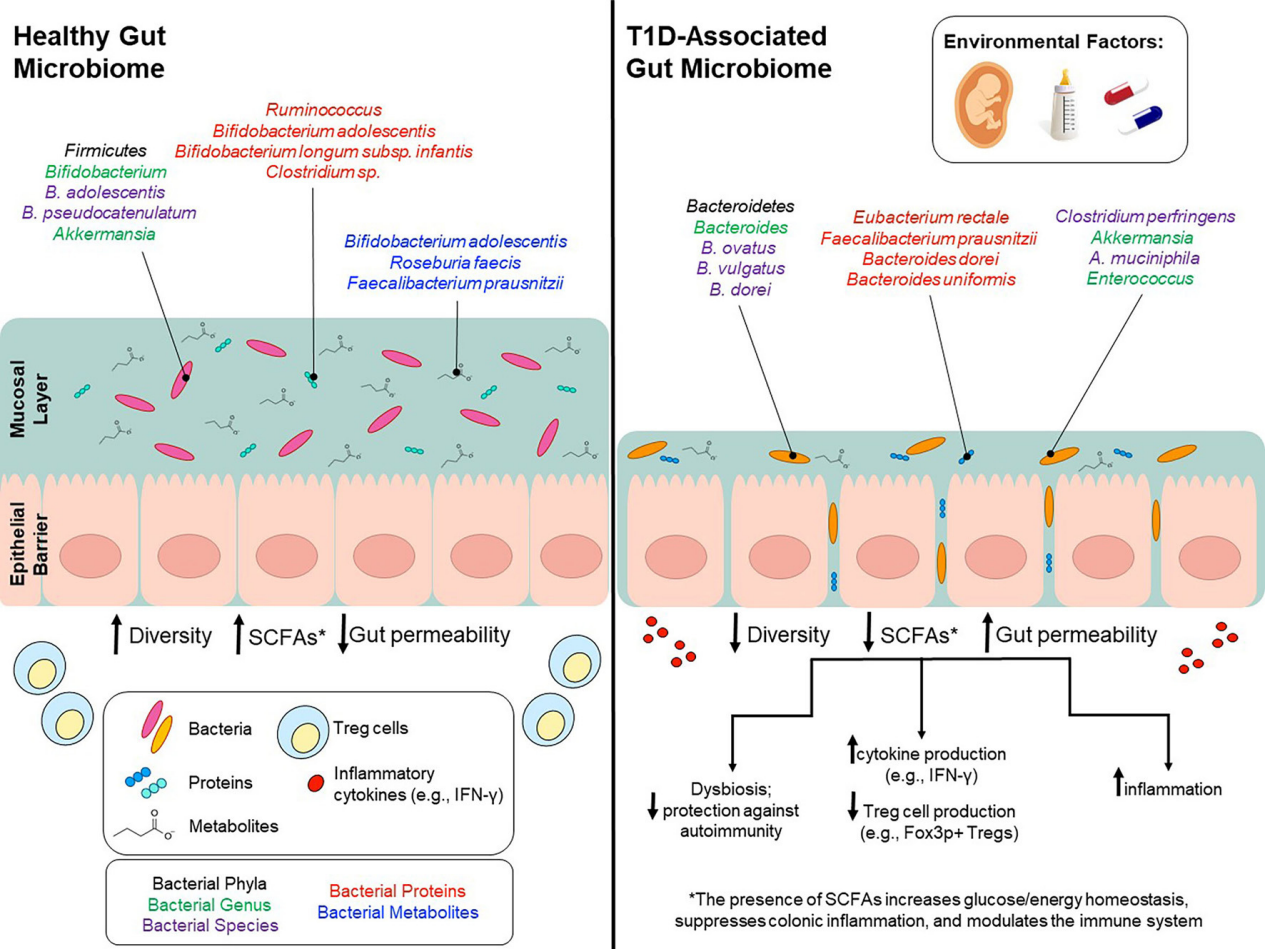
Environmental factors modulate gut microbiota and potentially contribute to T1D onset. Environmental factors, such as birth mode, diet early in life, and use of antibiotics can influence gut microbiota composition and can to lead to lower bacterial diversity, decreased SCFA production and increased gut permeability. Bacterial phyla/genus/species that are affected by environmental factors and differ between T1D patients and healthy controls are depicted in the colors black/green/purple, respectively. Bacterial genus/species that have been identified in proteomic analyses and are increased in either T1D patients or healthy controls are shown in red. Bacterial genus/species that have been identified in metabolomics analyses and are increased in either T1D patients or healthy controls are shown in blue.

Starting in 2008, DIABIMMUNE recruited ~1,000 newborn infants with high-risk HLA haplotypes from Finland, Estonia, and Russia. Blood and stool samples, along with clinical metadata, were collected from 1 month to 3 years of age. An initial analysis published in 2015 focused on the Finnish and Estonian subjects (75). This study examined a cohort of 33 infants genetically predisposed to T1D. Of this cohort, 11 children developed autoantibodies (seroconverted), and out of the 11 seroconverted subjects, four subjects developed T1D. Microbiome analyses were performed using 16S rRNA and metagenomic shotgun sequencing data. Overall, this study found a decrease in microbial diversity and a reduction in bacterial gene content in autoantibody-positive children during progression toward T1D. Specifically, they found a decrease in *Lachnospiraceae* and *Veillonellaceae* in children who developed diabetes, and an increase in *Streptococcus, Blautia, and Ruminococcus* genera (Table 1, Figure 1). A functional analysis found that bacterial metabolism in autoantibody-positive subjects is characterized by a higher prevalence of genes involved in sugar transport and a lower prevalence of genes associated with amino acid biosynthesis compared to subjects that did not seroconvert.

In a subsequent DIABIMMUNE study (76), a metagenomic analysis of 222 Finnish, Estonian, and Russian children (sub-cohorts of 74 children from each country), matched for HLA risk and gender, found no correlation between islet autoantibodies or T1D status and microbiome composition. However, the authors reported differential abundance of *Bacteroides* species. *Bacteroides* species were lowly abundant in Russian children compared to neighboring Finland and Estonia. The authors postulate that the higher incidence rates of T1D in Finland and Estonia are associated with early life lipopolysaccharide (LPS) exposure. LPS exposure would potentially arise from *Escherichia coli* for Russian children whereas children from Finland and Estonia would more likely have early-life exposure to *Bacteroides* species' LPS.

As with TEDDY, DIABIMMUNE identified differences in gut microbiota composition and an overall decrease in microbial diversity in T1D patients. This study also demonstrated a potential mechanism using *in vitro* experiments which elucidated the structural and functional characteristics of *Bacteroides dorei* LPS compared to *E. coli* LPS. The results of the latter study and the effects of early life LPS exposure is further discussed in the Breastfeeding section (Breast Milk and Its Role in Gut Microbiota Modulation).

### Abis Study

All Babies in Southeast Sweden (ABIS) is a prospective birth cohort to address the etiology of immune-mediated diseases, specifically T1D (83). All mothers in the Southeast of Sweden that gave birth between October 1997 and October 1999 were asked to participate. In total, ~17,000 babies participated in the study (78.6% of those born in the region), and to date, 147 of them went on to develop T1D. Blood, fecal and other biological samples were collected from newborns at 1, 2.5–3, 5–6, 8, and 11–12 years of age. In a recent study using the ABIS cohort, authors investigated the correlation between HLA haplotype and its effect on gut microbiota composition (23). Previous work in mice has shown that the inability to present class II antigens and/or different MHC class II haplotypes results in differences in gut microbiota composition (23, 84–86). In one study, the lack of class II antigen presentation led to a decrease in *Lactobacillus* species and an increase in segmented filamentous bacteria (85). In the ABIS study, Russell et al. (23) found similar results in humans in which the genetic risk for developing T1D autoimmunity was associated with alterations in gut microbiota composition. They discovered that the core gut microbiota composition and the beta diversity differed depending on the HLA risk group and overall genotype. Moreover, certain protective HLA haplotypes were correlated with the genera *Intestinbacter* and *Romboutsia* (23). Another interesting study examined the relationship between exposure to pets and T1D. They found that 45.5% of pregnant women recruited for the study owned pets. Most common were cats and dogs, and neither exposure to dogs nor cats were associated to T1D risk (87). However, they found exposure to hamster's significantly increased T1D risk. This is a curious finding since a recent study based on the Canadian Healthy Infant Longitudinal Development Study (CHILD) showed that early exposure to household pets alter the gut microbiota composition of the infants which may reduce the risk of some of the diseases including obesity and allergic diseases (88).

## Environmental Factors Impacting Gut Microbiota and T1D

Between birth and age 3, the infant microbiota changes drastically due to its constant exposure to novel environmental/maternal microbes and food/animal-born antigens. By age 3, these fluctuations diminish as the microbiota composition settles into its adult-like state (74). Early life factors, such as birth method, breastfeeding, exposure to cow's milk, and introduction to solid foods has been shown to influence early-life microbiota composition, thereby dictating its successional composition. Thus, early life factors have consequences beyond infancy and most likely influence health and disease development later on in life. In the following section we will discuss (i) how environmental factors influence microbiota composition, (ii) how environmental factors influence T1D risk, and (iii) the interplay between environmental factors, gut microbiota, and T1D onset and progression.

### Birth Method

Previous research has implicated that the uterus is a sterile environment, completely devoid of bacteria. However, current research efforts have called this into question. A number of studies have found that during the intrauterine period of development, the fetus is exposed to maternal microbiota via transplacental passage into the amniotic fluid (89). Pre-partum, unidirectional shifts in the maternal gut microbiota have also been observed between the first and third trimester, presumably influenced by hormonal and immunologic changes during this period of time (90). Interestingly, this shift enriches for butyrate-producing taxa that promote increased levels of immunomodulatory T regulatory cells (90). This is thought to reduce the chances of maternal rejection of the fetus (90). A second shift in gut microbiota is then observed just prior to giving birth. This shift leads to increased heterogeneity, reduced alpha diversity, and an overrepresentation of taxa that are known to induce inflammation (90, 91). Studies in humanized germ-free mice have suggested that these changes are adaptive and promote maximum energy transfer between mother and fetus (91). These taxa are also thought to promote the successful colonization of anaerobic species that dominate the neonate's early life microbiota. The mother's vaginal microbiota also undergoes alterations just prior to delivery. During pregnancy, four major *Lactobacillus* species increase in abundance and contribute to the stability of the community while also lowering the alpha diversity (90). These dominant taxa do not play a significant role in maintaining the vaginal pH or in preventing infection, but instead are thought to increase in abundance due to their importance in initial neonate colonization (90).

#### Birth Method and Its Role in Gut Microbiota Modulation

A number of studies have correlated birth method with distinct variations in neonatal microbiota (92–96). Links between birth mode and an increased risk of obesity, asthma, allergies and autoimmune diseases have also been reported (96–105). Taken together, the evidence strongly suggests that delivery mode has a profound impact on microbiota composition and succession. Vaginally-delivered infants have a microbiome most similar to the vaginal microbiome of the mother while C-section-delivered babies are typically colonized with species found on the mother's skin (96). Differences in taxa are most distinct during the first 3 months of life. The microbiome of infants delivered vaginally are initially dominated by commensal bacteria, such as *Lactobacillus* and *Bifidobacterium* while infants born by C-section are predominantly colonized by *Clostridium* species and skin bacterial communities, most notably, *Staphylococcus* species (96). TEDDY also found that children delivered vaginally had a higher level of *Bacteroides* which, in turn, was linked to higher levels of diversity and faster maturation of the gut microbiota (74).

The first stages of microbial exposure/colonization ultimately determines the composition and succession of the infant's microbiota and influences overall metabolism and immunomodulation. Thus, early colonization by aberrant microbiota can have long-term effects on immune function and can increase a child's risk for developing an autoimmune-related disease, such as T1D. Several studies have found that the colonization of *Lactobacillus, Bifidobacterium*, and *Bacteroides* is delayed or completely absent in infants born via C-section (92, 96, 106, 107) and the lack of exposure to vaginally-derived *Lactobacillus* species can lead to differences in microbial succession. Subsequent diversity for the major phyla of gut microbiota—Actinobacteria (dominated by *Bifidobacterium*), Bacteroidetes, and Proteobacteria—is also significantly lower in C-section-delivered infants (92, 107–112).

#### Birth Method and T1D Onset

A 2008 meta-analysis by Cardwell and colleagues compared the results of 20 different studies to determine if T1D risk is correlated with birth mode. After adjusting for covariates, including birth weight, gestation time, maternal age, maternal T1D incidence, and breastfeeding, there was still a 20% increased risk of T1D onset in infants born via C-section (113). Yet, taking individual research results into consideration reveals a number of discrepancies. Several studies have observed a significant correlation between birth mode and T1D risk (113–116) while others have revealed little to no association (103, 116–118).

Research into the direct link between birth mode, microbiota and T1D incidence is scarce; nevertheless, evidence to date suggests that C-section delivery and aberrant microbiota has an influence on T1D onset and progression. In an NOD mouse study, researchers identified distinct differences in microbiota composition between C-section and vaginally delivered pups although they did not find a significant correlation between birth method and T1D incidence, (119). They also observed lower levels of Foxp3+ T-regulatory cells, anti-inflammatory IL-10, and tolerogenic CD103+ dendritic cells, suggesting that C-section delivery has long-term effects that potentially lead to dysfunctional immunosuppression, thereby, increasing the risk of developing anti-islet autoimmunity (119).

Further analyses into the specific genera implicated in disease onset reveals striking inconsistencies in the role that Bacteroidetes plays. As previously mentioned, neonates delivered via C-section have a decreased abundance of *Bacteroides* species. *Bacteroides* play an important role in overall immune system development and function by stimulating the plasmocyte production of secretory IgA (116, 120). More specifically, *B. thetaiotaomicron* is involved in maintaining the gut barrier while *B. fragilis* and *B. subtilis* have been shown to promote gut-associated lymphoid tissue maturation and aid in pre-immunity antibody production (116, 121–123). *B. fragilis* also inhibits the pro-inflammatory cytokine IL-17 in the intestine (116, 124).

Conversely, one of the most notable distinctions in microbiota for seroconverted high-risk children is the increased abundance of *Bacteroides*. One example is the longitudinal T1D Prediction and Prevention study (DIPP); a Finnish research effort that started collecting stool and blood samples from children with the high-risk HLA genotype in 1994 (125, 126). A more recent study used four matched case-control pairs from DIPP to identify microbiota that differed between cases and controls (127). Based on their analyses, one major distinction was the abundance of Bacteroidetes and Firmicutes. Cases had a significantly higher abundance of Bacteroidetes, which increased over time, compared to controls. In support of these results, amongst the taxa identified in the DIABIMMUNE study, *Bacteroides* species were found to be most abundant in the seroconverted cohorts from Finland and Estonia (76). Similar studies have found that *Bacteroides* species were overrepresented in case subjects; specifically, the species *Bacteroides ovatus*, which was responsible for 24% of the observed increase (127). A more recent Finnish study (128) corroborates these findings using a cohort of 76 at-risk children which they followed from birth to 2 years of age. Metagenomic analyses identified two *Bacteroides* species, *B. dorei* and *B. vulgatus*, that were significantly higher in cases compared to controls prior to seroconversion (128). However, a number of studies have come to different conclusions or have found no correlation at all between *Bacteroides* abundance and T1D onset (129). Table 1 and Figure 1 summarize the findings of these studies and the differences in the microbial composition of T1D patients and the controls.

### Breastfeeding

Extensive research has elucidated the many benefits breast milk provides for an infant's growth and development (130–133). Bioactive agents contained in breast milk, including antimicrobial and immunomodulatory components, have been shown to modulate gastrointestinal and immune function along with gut microbiota composition through a multitude of mechanisms (130, 131, 133, 134).

#### Breast Milk and Its Role in Gut Microbiota Modulation

Alongside bioactive compounds, the maternal microbiota is transferred in breast milk and changes in the infant gut microbiota are correlated with breastfeeding frequency in a dose-dependent manner (135). Specifically, lactic acid bacteria in the genera *Bifidobacterium* and *Lactobacillus*, capable of breaking down human milk oligosaccharides (HMOs), are transferred from mother to child (132, 136, 137). These genera are known to maintain the intestinal barrier, stimulate the production of IgA antibodies and are involved in the production of short chain fatty acids (SCFAs) (132, 138–144). Of note, the species *Bifidobacterium longum* subsp. *infantis* is enriched in infants that are strictly breastfed the first 6 months of life (74). *B. infantis* is particularly efficient at metabolizing HMOs into SCFAs (indirectly) and has also been shown to promote mucus production, alleviate diet-induced colonic mucus deterioration, and play an important in immuno-regulation (145, 146) (Figure 1).

#### Breast Milk and T1D Onset

Successful immunologic development consists of interactions between the host and its microbes at the mucosal surface of the intestine (145, 147). Murine model studies have identified critical developmental windows in which successful microbe-driven immuno-regulation can occur. The same is shown to be true in humans as discussed in the Diet section Gluten-Based Foods. These data suggest that the microbes present and the timing of introduction are key to proper immune system development. For children genetically predisposed to T1D, breast milk is thought to contain protective properties against T1D development (148, 149). Moreover, infants that are strictly breastfed the first 6 months of life have a distinct microbiota composition. It is postulated that the protective properties of breast milk work by modulating gut microbiota composition (94, 132, 150–155).

In a 2015 Norwegian study (MIDIA) investigating the association between breastfeeding duration, age of introduction of solid foods, and the risk of islet autoimmunity/T1D in genetically susceptible children found that any frequency of breastfeeding for 12 months or longer was correlated with a slower progression toward, and an overall decrease in, T1D incidence (156). However, neither the age of solid food introduction nor whether infants were being breastfed at the time of introduction had any effect on islet autoimmunity and/or T1D progression (156). These results indicate that breastfeeding alone has a significant impact on T1D progression/onset while the introduction of other food sources, such as solid foods, may not play as significant of a role (157–159).

Microbiota studies have often looked to the Firmicutes:Bacteroidetes ratio as a potential harbinger for disease development. Although the correlation between disease and shifts in higher-order ratios is currently speculative, similar shifts between *Bifidobacterium* and *Bacteroides* have been observed in children at-risk for T1D. Since both genera metabolize HMOs, this inverse correlation is thought to stem from interspecific competition for a common resource (160, 161). Interestingly, these shifts are paralleled in comparisons between breastfed (more *Bifidobacterium*) and formula-fed (more *Bacteroides*) infants. Perhaps unsurprisingly, an inverse correlation between *Bifidobacterium* colonization and T1D disease development has been observed in a number of cross-sectional and longitudinal human studies (145, 161). Moreover, Mejía-Leon et al. discovered an enriched population of *Bacteroides* in recently diagnosed type 1 diabetics (162). Similarly, a higher incidence of T1D in Finnish and Estonian populations has been documented alongside a higher prevalence of *Bacteroides* in at-risk children when compared to at-risk children in nearby Russia (76).

In the second DIABIMMUNE study discussed above, authors showed that *Bacteroides* species, such as *B. dorei*, inhibit immune stimulation and inflammatory cytokine responses to *E. coli* which results in suppressed innate immune signaling and a lowered endotoxin tolerance (161). This immune suppression is modulated by high levels of *Bacteroides-*derived lipopolysaccharides (LPS) that are structurally and functionally distinct from those produced by *E. coli;* the dominant form of LPS present in Russian infants (161). Moreover, *in vitro* experiments show that LPS produced by various taxa can either inhibit or trigger TLR4, NF-κB activation, and endotoxin tolerance (163–167). Specifically, when NOD mice were injected with an immunogenic LPS derived from *E. coli*, endotoxin tolerance was elicited in conjunction with a decreased incidence of T1D (161). *B. dorei* LPS did not provide the same protection against disease onset (161). These results support previous NOD mouse studies in which LPS were found to have a direct impact on T1D progression (161, 168). The mechanisms involved in LPS-modulated immunity and its link to T1D development are not fully understood, but previous work by Gulden et al. (169), and Wen et al. (68) has revealed the importance of toll like receptor-3 (TLR3) and Innate Immune Signal Transduction Adaptor (MyD88) in T1D onset in NOD mice; two components of the LPS/TLR4 signal transduction pathway (68, 161, 169).

Taken together, results derived from both *in vivo* and human studies have elucidated the importance of certain breast milk taxa in immune system development and modulation. Some genera, such as *Bifidobacterium*, play an important role in the overall health and development of an infant, and have also been shown to play a specific role in protecting at-risk children against T1D onset (Figure 1). However, in recent studies, even strictly breastfed infants were void of *Bifidobacterium* species, suggesting that mothers are not being colonized by these genera to the same extent as their predecessors. In one U.S. study, 30% of strictly breastfed infants had no detectable *Bifidobacterium*, and for those infants with *Bifidobacterium* colonization, only 30% had a microbiota in which *Bifidobacterium* represented over 50% of the population (145, 170).

### Dietary Factors

The overall incidence of T1D has increased considerably over the past half of the twentieth century (171, 172). Current evidence has pointed toward dietary factors as important contributors to T1D development. Some factors have been suggested to trigger or accelerate disease progression, while others protect against the development of T1D-related autoantibodies and overall disease progression (171, 173, 173–183). Both animal and human studies have indicated that early life exposure to foreign food antigens, such as gluten and bovine insulin can influence β-cell autoimmunity (184–186). However, how and to what extent these dietary factors affect disease outcome remains to be discovered.

#### Gluten-Based Foods

A common physiological feature of T1D pathogenesis is a weakened intestinal barrier (i.e., leaky gut syndrome) which contributes to increased inflammation in T1D patients (38, 187). Gluten, which consists primarily of monomeric gliadin and polymeric glutenins, can also contribute to gut permeability and induce inflammation by triggering the release of cytokines (188–191). Thus, intestinal microflora that help regulate intestinal barrier function are crucial for dampening the pro-inflammatory response. Gluten-based diets are thought to contribute to gut permeability through the modulation of gut microbiota (192–194). In support of this, taxa associated with a gluten-free diet, such as *Akkermansia* species, have been shown to regulate human epithelial tight junction proteins, protect against pathogen invasion, and play a protective role against T1D onset (195, 196).

##### Gluten-based foods and T1D onset

Thus far, the data on the effect of gluten-based foods in humans has shown a wide range of variance. A number of studies have found that placing patients on a gluten-free diet does not markedly improve their autoantibody titers (i.e., reduce). However, these same studies have also shown that a gluten-free diet can improve an individual's insulin response in a glucose tolerance test (197, 198). A few studies focused on patients with both coeliac disease and T1D observed a significant improvement in overall health and diabetic control in subjects provided with a gluten-free diet (193, 199). Nonetheless, other studies have not observed such affects (200, 201).

Despite these apparent inconsistencies, one factor has been ubiquitously identified as playing a crucial role in disease development; the timing and mode of gluten introduction. Human studies have found that early exposure (<3 months of age) to gluten increases the risk of islet autoimmunity and that this risk is mitigated if gluten is introduced while the child continues to breastfeed (51, 202–204). This trend is mirrored in animal studies (205) and has been found to affect T1D onset in a dose-dependent manner (206, 207).

The diet of the mother during pregnancy has also been investigated in relation to a child's T1D risk. When NOD mice mothers were fed a gluten-free diet, the incidence of T1D in their offspring was significantly reduced (208, 209). One such study observed an astounding 7-fold+ decrease in T1D incidence in NOD pups (62.5–8.3%) (209). However, the results from human studies have not been so consistent. A recent study by Antvorskov et al. (210) found that in a cohort of Danish women, the risk of developing T1D was directly proportional to the maternal gluten intake during pregnancy (210). Women that consumed the highest amount of gluten (>20 g/day) had double the risk of T1D onset in their children. In contrast, two previous studies found no strong correlation between a mother's gluten intake and T1D incidence in the child (211, 212).

Early life exposure to diabetogenic dietary factors has a major influence on T1D onset. Even so, eliminating gluten from the diet of older children diagnosed with T1D has been shown to improve diabetic metrics including prolonged remission periods and reduced HBA1c (213, 214). A similar study reported improved glucose tolerance and insulin sensitivities, but no improved titers in islet autoantibodies in older children placed on a gluten-free diet for 6 months (median age = 16 years old) (197).

Overall, these research efforts have provided vital information on the role that gluten plays in T1D onset. Nonetheless, human-subject longitudinal studies are needed to make the connection between diet, its influence on the gut microbiome, and how this effects T1D onset and progression. One such study is the German longitudinal cohort study, BABYDIET study.

##### BABYDIET study

BABYDIET, a substudy of the larger BABYDIAB study, was initiated to determine if early-life gluten exposure plays a role in T1D onset. This study followed 22 case children displaying autoimmunity along with 22 matched controls for a little over 3 years. Stool samples were collected monthly between ages 3 and 36 months, and at 6-month intervals thereafter. All subjects had at least one first-degree relative with T1D. Overall, no significant differences were observed between cases and control for microbial diversity (i.e., richness, evenness) and composition once data was corrected for multiple comparisons (215, 216). However, when comparing the overall microbiota network [i.e., Eigenvector Centrality (EC), Node Degree] of children displaying autoimmunity vs. autoantibody-negative children, significant variations emerged. Autoantibody-positive children showed markedly different centrality distributions compared to healthy controls at both 6 months and 2 years of age. There were also more network nodes that had intermediate levels of connectivity in the autoantibody-positive cohort. Furthermore, specific genera had variations between cases and controls in an age-dependent manner. For example, at 6 months of age, the bacterial genera *Enterococcus, Sarcina, Prevotella*, and *Corynebacterium* showed high EC in networks of children who became autoantibody positive. In contrast, genera that displayed high EC at 2 years of age in autoantibody-positive children include *Barnsiella* and *Candidatus Nardonella*, whereas *Staphylococcus* and *Nocardioides* had high EC in the autoantibody-negative group. It is worth noting that these observations were in the genera level as a consequence of 16S sequencing.

#### Cow's Milk and Cow's Milk Proteins

Animal model studies and human studies alike have found associations between T1D incidence and the consumption of cow's milk at both weaning and later in development. Retrospective meta-analyses on national dietary consumption records have been used to determine if national and/or global patterns of cow's milk consumption correlates with regional T1D incidence rates (184, 217, 218). Several analyses performed by Muntoni and colleagues found a positive association between T1D and a region's intake of animal-based energy along with a region's overall milk supply (217). A meta-analysis specifically comparing a nation's T1D incidence rate with its annual cow milk protein consumption also found a positive correlation between a nation's T1D incidence rate and its annual milk protein consumption (184, 218). A negative association between breastfeeding (at 3 months) and T1D risk was also observed (184).

The Finnish Diabetes Prediction and Prevention Project (DIPP) studied general food consumption in children with advanced β-cell autoimmunity and found that the general intake of cow's milk products was one of the few factors directly associated with β-cell autoimmunity. This was not true of other dairy products, such as sour milk products and cheese which suggests that the increased risk of β-cell autoimmunity comes specifically from cow's milk-associated proteins (219). Looking more closely at the immune response to these proteins, several studies have observed elevated levels of cow's milk-specific IgG and IgA antibodies in early onset T1D (220–222). Interestingly, Elliott and colleagues found that the total protein consumption was not correlated with T1D incidence, but the specific consumption of cow's milk-derived β-casein (A1 variant) was (218). These results indicate that an elevated humoral response is mounted against cow's milk proteins in T1D patients, making them a potential contributor to the autoimmune process leading up to T1D onset (222).

While evidence supports a role for cow's milk proteins in T1D onset, other studies have found no such correlation (223). Conversely, other studies have suggested that cow's milk-based formula plays a protective role against T1D incidence (224). Human studies investigating the relationship between cow's milk proteins, gut microbiota composition and T1D derive from two major longitudinal studies. These dietary studies include: (1) the Trial to Reduce IDDM in the Genetically at Risk (**TRIGR**) and (2) the Finnish Dietary Intervention Trial for the Prevention of T1D (**FINDIA**). While these studies do not specifically address the association between the gut microbiome and T1D incidence, a cross-sectional study derived from these studies was subsequently conducted to investigate this affect.

##### Trial to reduce IDDM in the genetically at risk (TRIGR)

In the TRIGR study, exposure to foreign dietary proteins was postponed until 6–8 months of age and the development of autoantibodies (insulin, GAD, IA-2, and ZnT8) was monitored until the age of 6 years (172). The aim of this study was to test whether supplementing breast milk with highly hydrolyzed milk formula is able to inhibit T1D onset. Infants (*n* = 230) with confirmed HLA-associated susceptibility and at least one family member with T1D were enrolled in the study. Subjects received either casein hydrolysate formula or conventional formula whenever breast milk was not available and were subsequently followed up to age ten. Over the course of the study, 17 children developed at least one autoantibody in the casein hydrolysate group (17%) compared to the 33 children in the control group (30%). Eight children in the casein hydrolysate group (8%) and 17 (16%) in the control group developed two or more autoantibodies. Insulin-autoantibodies were the most frequently observed first-autoantibodies with anti-islet autoantibodies being a close second. Overall, this study found weak associations between formula intervention and T1D development. A more recent update on this study published in 2018 found that weaning to hydrolyzed formula did not reduce the risk of T1D in children with an increased disease risk up to the median age of 11.5 years (225).

##### Finnish dietary intervention trial for the prevention of T1D (FINDIA)

FINDIA is a multisite double-blind clinical trial in which newborn infants were randomized to receive a bovine insulin-free cow's milk formula (CMF), a whey-based hydrolyzed formula (WHF), or a whey-based FINDIA formula (FINDIA group) from which bovine insulin was removed. Between 2002 and 2005, subjects were recruited from three pediatric hospitals in Finland. One hundred thirteen infants with HLA-conferred susceptibility to T1D were randomly assigned to receive one of the three formulas. Results from the FINDIA study (77) suggest that administering bovine insulin-free formula during the first 6 months of life reduces a child's risk of developing β-cell autoantibodies by age three. When the microbiota of seroconverted subjects were compared to non-seroconverted subjects, researchers found an increased abundance of *Bacteroides*, and a decreased abundance of *Bifidobacterium*. In the intention-to-treat analysis, 6.3% of children in the CMF group, 4.9% in the WHF group, and 2.6% of children in the FINDIA formula group were positive for at least one autoantibody by age three. The researchers found that compare to ordinary formula, weaning to an insulin-free formula (FINDIA group) reduced the cumulative incidence of autoantibodies by age 3 years in children at genetic risk of T1D.

##### TRIGR-FINDIA cross-sectional Study

In a study by de Gaffau et al. (36), gut microbiota composition was analyzed and compared between autoantibody-positive (*n* = 18; tested positive for at least two autoantibodies) and matched autoantibody-negative (*n* = 18) children. Subjects were recruited from TRIGR and FINDIA. Compared to autoantibody-negative children, autoantibody-positive children had higher relative abundance of *Bacteroidetes* (*Bacteroides* genus). However, at the species level, there were only a few *Bacteroides* species associated with autoimmunity, alongside a higher abundance of various *Firmicutes*, such as *Clostridium perfringens*; both of which are known to play a role in increased gut permeability. This analysis also suggests that low abundances of *Bifidobacterium adolescentis* and *Bifidobacterium pseudocatenulatum* (<12% combined) are significantly correlated with increased levels of beta-cell autoimmunity (Figure 1).

### Antibiotics

Antibiotics are used to control bacterial infections and have a range of effects on the gut microbiota. Antibiotic exposure has been found to significantly decrease bacterial diversity; however, several studies have shown that adult gut microbiota often recovers to resemble microbiota prior to treatment (226). Nevertheless, a 4-day intervention with a cocktail of three antibiotics (meropenem, gentamicin and vancomycin) caused the loss of nine bacterial species in adult men 180 days after antibiotic use (226). This same study also observed an initial bloom of pathobionts including *Enterococcus faecalis* and *Fusobacterium nucleatum* and a depletion of Bifidobacterium (226).

On average, a child in the U.S. receives three antibiotic courses before they are two, and 10 courses before they are 10 (227). Despite their common administration to infants and children, their effects on gut microbiota and how it relates to human diseases, such as T1D, is still not fully understood. In a study on infants, antibiotic exposure was associated with diminished *Clostridiales* and *Ruminococcus* in the first 3–9 months of life, and delayed microbiota maturation (228). In contrast to this, a large Norwegian Mother and Daughter Cohort study did not find an association with acetaminophen use and T1D risk. In this study, the use of antibiotics by the mothers and the child in the first 6–9 months was also not found to be correlated with T1D risk (229). Likewise, TEDDY found that frequency and use of antibiotics during the first 4 years of life did not influence the risk of developing autoimmunity for T1D or Coeliac Disease (230). A population-based case-control study that included all T1D cases of children born between 1997 did not find a correlation between antibiotic use and T1D. However, a positive association was seen between children who took broad-spectrum antibiotics in the first 2 years and T1D (231). In a population-based mother-child cohort that included all children born in Finland between 1996 and 2000 who were diagnosed with T1D, they found an increased risk of T1D in children of mothers who took phenoxymethyl penicillins or quinolone before pregnancy. They proposed that use of antibiotics by mothers may diminish the transfer of healthy microflora to the baby. A higher risk of T1D was found when the mother took macrolides before pregnancy and the child took macrolides compared to mother-child pairs where neither took macrolides. A high risk of antibiotics, defined as seven or more purchases of antibiotics, was also found to have an increased risk of T1D. *Bifidobacterium* and *Lactobacillus* species are associated with a healthy microflora and are particularly sensitive to macrolides, quinolines, and penicillins. The study proposes that macrolides and quinolones might increase the risk of T1D in children by preventing the synthesis of DNA and enzymes in β-cells resulting in beta cell death (232). Another case-control study used THIN, a UK population medical record database that includes complete medical records of about ten million patients, to look at the effect of antibiotic exposure on both type 1 and type 2 diabetes. The study found that exposure to a single antibiotic was not associated with higher diabetes risk. However, taking two to five antibiotic courses of penicillins, cephalosporins, macrolides, or quinolines was associated with an increase in diabetes risk, and taking more than five courses of quinoline was associated with an increased T1D risk. Exposure to more than five courses of penicillin was also associated with an increased diabetes risk (233).

Several studies have been performed on Bio-breeding Diabetes-Prone (BB-DP) rats, LEW1.WR1 rats, and NOD mice to determine the impact of antibiotic treatment on T1D incidence and the gut microbiome composition. In a study performed by Brugman et al. (192) male and female BB-DP rats on either a conventional plant based (CON) or hydrolyzed casein (HC) diet continuously received antibiotic treatment with broad spectrum antibiotics Bactrimel (sulfamethoxazole and trimethoprim) and colistine sulfate. Antibiotic treatment while on the CON diet was found to reduce T1D incidence and delay onset to 30 days whereas antibiotic treatment while on the HC diet was found to protect against T1D onset (192). Additionally, BB-DP rats that later developed T1D were shown to have higher abundance of *Bacteroides* when compared to controls and rats that did not develop T1D (192). Similarly, a 2012 Hara et al. study investigating LEW.WR1 rats infected with kilham rat virus (KRV) in order to induce T1D found that virus-induced T1D was suppressed by treatment with the antibiotic sulfatrim. Treatment with sulfatrim was also found to suppress the virus-induced anti-islet responses in the LEW1.WR1 rat via the down-modulation of the immune system. Furthermore, infection with KRV caused an increase in abundance of *Bifidobacterium* and *Clostridium* (234). Contrary to these studies, Livanos et al. found that pulses of therapeutic doses of the macrolide antibiotic tylosin tartrate (pulsed therapeutic antibiotics, PAT) accelerated T1D and insulitis development in male NOD mice. The study also found that the group receiving PAT had larger abundance of *Akkermansia* and *Enterococcus* when compared to control groups which were more abundant in *Bifidobacterium* (235). A 2012 study performed by Hansen et al. investigating the effects of early life treatment with the antibiotic vancomycin on NOD mice found that mice treated with vancomycin from birth until 28 days of age had a significantly lower incidence of T1D and higher clusters of CD4+ T cells producing pro-inflammatory cytokines. Conversely, NOD mice that received vancomycin from 8 weeks of age until onset of T1D had higher diabetes incidence, higher insulitis scores, and increased blood glucose levels, indicating that early life treatment significantly impacts T1D progression. Treatment with vancomycin was also seen to affect microbial diversity as *Akkermansia muciniphila* dominated the gut microbiome of all groups treated with vancomycin (236). In a similar study, the effects of antibiotic treatment on T1D progression in the offspring of NOD mice treated with antibiotics while pregnant further found the importance of antibiotic treatment early in life (237). The authors treated pregnant NOD mice with a combination of three antibiotics; neomycin, polymyxin B, and streptomycin, and found the offspring of treated mice to be significantly more protected than mice that received the antibiotic immediately after birth and mice that received antibiotic treatment at 3 weeks of age. The study illustrates the importance of early life or prenatal antibiotic treatment in order to combat T1D as the age the mice received treatment negatively correlated with protection from T1D. Additionally, the antibiotic treatment was seen to deplete gram negative proteobacteria and lead to dominance by gram positive bacteria of the *Firmicutes* family. According to the studies mentioned, it is unclear whether antibiotic treatment consistently increases or decreases T1D risk in animals, but it is always seen to have an effect on both microbial diversity and T1D incidence and there is evidence to suggest that early intervention is more effective.

## T1D and the Gut Microbiota: Looking Beyond Bacterial Species

The birth of sequencing altered the way by which scientists asked questions pertaining to both environmental and human associated microbiota and has enabled the use of big data in a way that was not previously possible. As sequencing technologies advance, more researchers are turning to such methodologies to investigate the association between the human microbiome and autoimmune disease. Currently, in T1D research, scientists have investigated differences present in the gut proteome, virome and metabolome of T1D patients. In this section, we will discuss the results and advancements made in these ‘omics related fields.

### Gut Proteome

16S and shotgun sequencing studies inform us regarding the microbial composition of the gut microbiome. On the other hand, proteomics studies are needed to understand which proteins are actively produced by these gut microbes. Few studies to date have been conducted to analyze the intestinal microbial proteome of T1D patients. However, in one such study Pinto and colleagues (238) investigated the differences in the intestinal microbial proteome between children with established T1D (3 children) compared with the proteome of healthy children (3 children). In control samples, bacterial proteins from *Bifidobacterium adolescentis, Bifidobacterium longum* subsp. *infantis, Ruminococcus, Collinsella aerofaciens, Coprococcus comes*, and *Clostridium sp*. were found to be the most abundant while proteins originating from *Eubacterium rectale, Faecalibacterium prausnitzii, Bacteroides dorei* and *Bacteroides uniformis* varied between control and case samples (Figure 1). More recently, one study (239) compared stool samples from newly-onset type 1 diabetes patients, islet autoantibody-positive, and low-risk subjects. Metaproteomic analysis was utilized to differentiate these stool samples into these three categories based on the presence of human (host-derived) and microbial-derived proteins. In patients with new-onset T1D, there was a significant reduction in microbial-associated host proteins that are responsible for maintaining the mucous barrier, microvilli adhesion, and exocrine pancreas. Thus, T1D patients had a higher prevalence of intestinal inflammation and decreased barrier function. A previous study, published in 2016, sets the stage for larger-scale metagenomics studies by demonstrating, on a smaller-scale, how specific gastrointestinal microbial communities and host-microbe phenotypic relationships are associated with onset of human disorders (18). Although the role of exocrine pancreas function in T1D onset remain unclear, they showed that exocrine pancreas enzymes, including amylase, carboxypeptidase CPA1 and CUZD1are less abundant in T1D patients (18). These initial results are promising and provides the field with an initial insight into how the T1D metaproteome differs in functionality compared to controls. Further research into this area will provide more information on disease progression and prevention methods.

### Gut Virome

Viruses have long been considered potential triggers of T1D onset and progression (240). However, much of the research to date has focused on the role that viral infections play in this process and the data have been inconclusive. More recently, researchers have begun to investigate the role of the gut virome. The gut virome is highly understudied as it relates to both states of health and disease, however, characterization of the intestinal virome from birth to the development of autoimmunity has potential to be an important component to understanding the etiology of T1D. Using the Finnish Diabetes Prediction and Prevention (**DIPP**) cohort, stool viromes were collected for 19 cases (who turned autoantibody positive before the age of 2 and later went on to develop T1D) and 19 matched controls at months 0, 3, and 6 before the onset of islet autoimmunity (241). Viruses made up to 2% of all identified sequences, with a major predominance of bacteriophages which were identified in 52 of the 96 samples (54.2%). Surprisingly, only 10.4% of samples contained one or more human viruses including parechovirus, bocavirus, annelovirus, enterovirus, and/or sapovirus. Kramna and colleagues also found no association between the gut virome and islet autoimmunity (241).

A similar study conducted in 2017 (242) examined stool samples from 11 children who developed serum autoantibodies associated with T1D (of whom, five developed T1D). In contrast with Kramna et al. (241), this study detected a significantly lower abundance of *Circoviridae*-related sequences (the group of viruses includes circoviruses) and lower virome diversity in T1D cases. Most importantly, the researchers found that multiple lines of evidence suggest that over time, the developmental progression of viromes significantly differed between children who developed autoimmunity and those who did not. The dynamic change in Shannon diversity over time was different between controls and T1D cases for *Microviridae, Myoviridae*, and *Podoviridae*. Additionally, the changes in richness over time differed between controls and cases for *Microviridae, Myoviridae*, and *Podoviridae*. The Random Forests analysis of age-discriminative contigs also suggested differences between cases and controls (242). This study reports virome changes occurred before seroconversion which suggests possible causality. Using virome capture sequencing methods, a more recent study characterized the gut virome of pregnant women with (*n* = 35) and without (*n* = 26) T1D throughout the Environmental Determinants of Islet Autoimmunity longitudinal study. Two viruses, picobirnavirus and tobamovirus, were more frequently observed in pregnant women with T1D, and the abundance of 77 viruses differed between the two maternal groups including 88 enterovirus B types (243).

Taken together, these data suggest that there are significant differences in the gut virome of T1D patients and progressors. However, caution should be taken in interpreting these results as very few studies with very small sample size have been conducted on this subject. As with any new avenue of interrogation, differences in methodology between studies may contribute to variation observed in the conclusions.

### Gut Metabolome

*In vivo* studies have demonstrated the importance of gut microbiota in the production of host metabolites (244–246). Of the metabolites known to be modulated and/or produced by gut microbiota, short chain fatty acids in particular have been shown to play a significant role in many aspects of general homeostasis. When dietary fiber is consumed, it is digested and subsequently fermented in the colon by gut microbiota into short chains fatty acids (SCFAs), such as acetate, propionate, and butyrate (246). Diets high in fiber alter gut microbiota composition by facilitating the growth of SCFA-producing species. Furthermore, a 4-fold increase in SCFAs has been observed in breast-fed infants compared to their formula-fed counterparts (247).

Diets high in fiber have also been shown to reduce systemic inflammation. A study by Trompette et al. (248) demonstrated that mice fed a diet high in fiber had higher levels of SCFAs and were protected against allergic inflammation of the lung (248). In humans, a decrease in dietary fiber has been directly connected to a decrease in the SCFA butyrate (249). Of the SCFAs, butyrate is thought to be particularly important. Amongst its multiple roles, it improves glucose and energy homeostasis, is an energy substrate for colonic epithelium and suppresses colonic inflammation and carcinogenesis. Butyrate also plays an essential role in modulating the immune response by inducing differentiation of regulatory T cells (Tregs), particularly Fox3p+ Tregs, and inhibiting inflammatory cytokines, such as IFN-γ (26, 246, 250–253). Interestingly, when NOD mice were fed a diet designed to increase acetate and butyrate levels they experienced a decrease in T1D incidence when compared to controls (254).

In the longitudinal studies TRIGR and FINDIA, SCFA-producing species including *Bifidobacterium adolescentis, Roseburia faecis, and Faecalibacterium prausnitzii* were negatively correlated with the number of autoantibodies detected (36). Thus, children positive for three or more antibodies had the lowest levels of SCFA-producing species. In agreement with this, the TEDDY study observed a higher prevalence of microbial genes associated with fermentation and SCFA biosynthesis in healthy controls. Interestingly, the taxon associated with these functions were not consistent across geographic regions suggesting that the function, rather than the taxa, is conserved amongst healthy children (19). Together, these results indicate that species capable of producing butyrate are potentially protective against autoantibody formation in at-risk children; more specifically, various *Clostridium* clusters capable of forming butyrate from acetate alongside *Bifidobacterium* species which can form butyrate via lactate metabolism (Figure 1).

Mirroring the results derived from animal models, human studies have demonstrated a metabolic shift in patients preceding autoantibody development, insulitis, and/or T1D. Sardinia has the second highest T1D incidence rate just below Finland, making it a good candidate for T1D studies (255). In one Sardinian-based study, Culeddu et al. (256) discovered that gut microbial metabolic products, such as *p-*cresol sulfate and phenylacetylglycine were involved in the classification of T1D patients^294^. Using data acquired through the Finland-based DIPP longitudinal study, serum metabolomic profiles of children who progressed to T1D were compared to controls that did not have autoantibodies and/or had not developed T1D. For individuals who progressed to T1D, reduced levels of succinic acid and phosphatidylcholine (PC) were observed at birth while reduced levels of triglycerides and antioxidant ether phospholipids were observed in follow-up serum samples. Increased levels of pro-inflammatory lysoPCs, reduced levels of ketoleucine and increased levels of glutamic acid were also amongst the alterations detected months before the documented seroconversion to autoantibody positivity (257). Interestingly, metabolic profiles partially normalized for T1D patients after seroconversion. This implies that autoimmunity is a later manifestation of an earlier alteration which may include disturbances in metabolic function (257).

## Closing Remarks

As demonstrated, extensive research has been done to gain a complete understanding of T1D etiology, onset and progression. What's largely apparent in this body of work is that T1D is a multi-faceted disease with no one “smoking gun”; no one simple answer. Furthermore, the methods used to investigate this complex disease may influence the outcomes observed. One major distinction are the conclusions gained from human studies compared to animal model studies. Although animal model studies have provided in-depth knowledge on the specific mechanisms at play, significant differences intrinsic to mouse/rat models force one to take caution when attempting to interpret findings to human health applications. For one, histological work has shown distinct differences in both the physiology of the pancreas in mouse models and the pathophysiological progression of T1D within the β-cell islets (258, 259). Moreover, epidemiological data on T1D demonstrates a slight male predominance of incidence rate before puberty, however, the incidence is twice as high in males compared to females between the ages of 15–39 (260–262). In mice, female NOD mice develop T1D at a much higher frequency than their male counterparts. One study showed that a number of *Bacteroides* genus correlations were sex-dependent. One specific example is *B. ovatis*, which was found to be positively correlated with autoantibody-positive male children while this same species was negatively correlated with female autoantibody-positive subjects (36). Thus, further study into these seemingly elusive differences is needed in order to determine if sex differences are indeed driven by intrinsic differences in gut microbiota. It is important to note that researchers have identified around 500 different therapeutics to prevent or reverse T1D in NOD mice model, none of these treatments have successfully translated to an effective tool for human T1D (263, 264). The differences between NOD mice model and human data indicate that it is time to think about alternative animal models for the T1D research and focus on human studies.

The advancement of sequencing technologies has opened up entirely novel avenues for microbiota exploration; an area of research that is just gaining its foothold in biomedical research. Thanks to these advancements, researchers have been able to explore the role of microbiota as it pertains to a number of chronic life-threatening diseases, including T1D. On the other hand, we are living in a bacteria-centric microbiota approach. As summarized in section T1D and the Gut Microbiota: Looking Beyond Bacterial Species, there are very few virome, proteome and metabolomics studies. More strikingly, we are not aware of any T1D study focusing on the phageome (265), fungal microbiome (266) and archeome (267), and metatranscriptome (268). Gut microbiota and its products are still a “dark matter” and our knowledge is quite limited.

Another limitation is being gut-centric in the microbiota studies. We are not aware of any nasal (269), skin (270) or vaginal microbiota studies (271) in the T1D field. These are other important mucosal surfaces for microbiota-host interaction and might have a role in the autoimmune diseases including T1D.

It is important to note that there is no causal relation identified yet with any human disease and microbiota. Instead of large screening studies, a parallel approach might be to focus on hypotheses based on specific mechanisms. We and others have demonstrated the importance of microbial interactions in shaping microbial communities through both contact-dependent and chemically-mediated mechanisms (272). Recent work also suggests that community function, rather than specific taxonomic composition, may play a more integral role in disease development (19). Taken together, perhaps future efforts should look further into specific community interactions and overall function to gain a better understanding of the varying disease etiologies. For instance, we recently hypothesized that the presence of microbes carrying an insulin-like peptide could instigate a molecular mimicry mechanism in T1D and have since discovered viral insulins (273, 274). We also showed the presence of the sequences of these viruses in human fecal and plasma samples.

To conclude, the available data suggest that there is a correlation between T1D onset and gut microbiota but we are far away to claim a causal link. Going forward, mechanism-based investigations beyond taxonomic characterization has potential to be the key in elucidating the mechanisms at play between gut microbiota, the immune system and T1D pathogenesis.

## Author Contributions

SD contributed intellectually to the conceptualization, literature review, writing and editing of the review, contributed to all sections of the review including tables and the figure. BS contributed intellectually to the conceptualization, literature review, writing and editing of the longitudinal studies along with the tables, figure, and proteomics section. QH contributed to the introduction. CB, TY, CC, CR, and MF contributed to the environmental factors sections. JL contributed intellectually to the review, providing feedback and edits along with insight into the field of T1D research. BM contributed intellectually to the review, providing feedback and edits. EA provided supervision and contributed intellectually to the conceptualization, writing and editing of the review.

### Conflict of Interest

The authors declare that the research was conducted in the absence of any commercial or financial relationships that could be construed as a potential conflict of interest.
