# Supplementary material for: The Role of Gut Microbiota and Environmental Factors in Type 1 Diabetes Pathogenesis
Source: Front Endocrinol (Lausanne). 2020 Feb 26;11:78. doi: 10.3389/fendo.2020.00078 (PMC7057241; doi:10.3389/fendo.2020.00078)
Supplement: Supplementary file 1 [file Table_1.pdf]

Supplementary Table 1a. Taxa identified in longitudinal studies as increased in seroconverted subjects

Phylum	Class	Family	Genus	Species	Study Cohort	Reference
Firmicutes	Bacilli	Streptococcaceae	*Streptococcus*	*mitis*	TEDDY	Vatanen et al., 2018
Firmicutes	Bacilli	Streptococcaceae	*Streptococcus*	*oralis*	TEDDY	Vatanen et al., 2018
Firmicutes	Bacilli	Streptococcaceae	*Streptococcus*	*pneumoniae*	TEDDY	Vatanen et al., 2018
Firmicutes	Bacilli	Erysipelotrichaceae			TEDDY	Stewart et al., 2018
Bacteroidetes	Bacteroidales	Bacteroidaceae	*Bacteroides*		TRIGR-FINDIA	de Goffau et al., 2013

Supplementary Table 1b. Taxa identified in longitudinal studies as decreased in seroconverted subjects

Phylum	Class	Family	Genus	Species	Study Cohort	Reference
Actinobacteria	Actinobacteria	Bifidobacteriaceae	*Bifidobacterium*		FINDIA	Vaarala et al., 2012
Actinobacteria	Actinobacteria	Bifidobacteriaceae	*Bifidobacterium*	*adolescentis*	TRIGR-FINDIA	de Goffau et al., 2013
Actinobacteria	Actinobacteria	Bifidobacteriaceae	*Bifidobacterium*	*pseudocatenulatum*	TRIGR-FINDIA	de Goffau et al., 2013

Supplementary Table 1c. Taxa identified in longitudinal studies as increased in healthy control subjects

Phylum	Class	Family	Genus	Species	Study Cohort	Reference
Firmicutes	Bacilli	Lactobacillaceae	*Lactobacillus*	*rhamnosus*	TEDDY	Vatanen et al., 2018
Actinobacteria	Actinobacteria	Bifidobacteriaceae	*Bifidobacterium*	*dentium*	TEDDY	Vatanen et al., 2018
Firmicutes	Bacilli	Streptococcaceae	*Streptococcus*	*thermophilus*	TEDDY	Vatanen et al., 2018
Firmicutes	Bacilli	Streptococcaceae	*Lactococcus*	*lactis*	TEDDY	Vatanen et al., 2018
